# New Insights into the Role of Peroxisome Proliferator-Activated Receptors in Regulating the Inflammatory Response after Tissue Injury

**DOI:** 10.1155/2012/728461

**Published:** 2012-02-29

**Authors:** Miriam D. Neher, Sebastian Weckbach, Markus S. Huber-Lang, Philip F. Stahel

**Affiliations:** ^1^Department of Orthopaedic Surgery, University of Colorado Denver, School of Medicine, Denver Health Medical Center, 777 Bannock Street, Denver, CO 80204, USA; ^2^Department of Trauma and Reconstructive Surgery, University Hospital Ulm, Steinhövelstraße 9, D-89075 Ulm, Germany; ^3^Department of Neurosurgery, University of Colorado Denver, School of Medicine, Denver Health Medical Center, 777 Bannock Street, Denver, CO 80204, USA

## Abstract

Major trauma results in a strong inflammatory response in injured tissue. This posttraumatic hyperinflammation has been implied in the adverse events leading to a breakdown of host defense mechanisms and ultimately to delayed organ failure. Ligands to peroxisome proliferator-activated receptors (PPARs) have recently been identified as potent modulators of inflammation in various acute and chronic inflammatory conditions. The main mechanism of action mediated by ligand binding to PPARs is the inhibition of the nuclear transcription factor NF-**κ**B, leading to downregulation of downstream gene transcription, such as for genes encoding proinflammatory cytokines. Pharmacological PPAR agonists exert strong anti-inflammatory properties in various animal models of tissue injury, including central nervous system trauma, ischemia/reperfusion injury, sepsis, and shock. In addition, PPAR agonists have been shown to induce wound healing process after tissue trauma. The present review was designed to provide an up-to-date overview on the current understanding of the role of PPARs in the pathophysiology of the inflammatory response after major trauma. Therapeutic options for using recombinant PPAR agonists as pharmacological agents in the management of posttraumatic inflammation will be discussed.

## 1. Introduction

Severe trauma still represents the most frequent cause of death in people below the age of 40 years [1]. Despite research efforts and improved intensive care of patients with trauma, no causal protective therapy is currently available, and the clinical outcome of multiply injured patients is still poor. Following severe tissue injury, a series of inflammatory events is initiated that have to be tightly orchestrated to guarantee efficient tissue repair. A controlled posttraumatic inflammatory response consists of limited release of proinflammatory mediators and recruitment of immune cells that contribute to wound healing and restoration of organ function [2]. If trauma is severe or if additional “second hit” insults occur, this primary protective activation of the immune system may turn to an uncontrolled inflammatory response which might cause additional tissue damage and impair the outcome of trauma [3, 4]. In addition, early posttraumatic hypoxia and hypotension due to the interruption of the blood flow may induce ischemia and reperfusion injuries and increase the extent of harmful events [5]. 

Peroxisome proliferator-activated receptors (PPARs) are nuclear membrane-associated transcription factors which have recently been shown to possess profound anti-inflammatory functions in a broad field of injury-associated conditions including trauma of the central nervous system (CNS), ischemia/reperfusion injury, sepsis, and shock [6–8]. The present review outlines the current understanding of PPAR-mediated anti-inflammatory mechanisms and discusses both opportunities and limitations of PPAR ligands as potential treatment strategy in trauma. 

## 2. Peroxisome Proliferator-Activated Receptors (PPARs)

PPARs are ligand-activated membrane-associated transcription factors belonging to the nuclear hormone receptor family. To date, three subtypes of PPARs have been identified in various species, PPAR*α*, PPAR*β*/*δ*, and PPAR*γ*, that exhibit differential tissue distributions and ligand specificities [9, 10]. Whereas PPAR*β*/*δ* is ubiquitously expressed in a wide range of cells, PPAR*α* is found in tissues with high fatty acid catabolism such as brown adipose tissue, liver, heart, kidney, and skeletal muscle, and PPAR*γ* is mainly expressed in the brown and white adipose tissue [10]. A wide range of natural and synthetic compounds can function as PPAR ligands. The natural agonists include fatty acids and fatty acid derivates, mainly eicosanoids, that can bind to and activate all three PPAR subtypes. With respect to the synthetic ligands, fibrates as lipid-lowering drugs function as PPAR*α* agonists, and glitazones, a class of oral antidiabetic agents, have been described to bind to PPAR*γ* [9, 11]. 

PPARs are activated by heterodimerization with the retinoid-X receptor (RXR) into biologically active transcription factors. PPAR-RXR heterodimers then bind to specific DNA sequences, known as peroxisome proliferator response elements (PPREs), in the promotor region of target genes, thereby acting as a transcriptional regulator [12]. In addition, PPAR*α* and PPAR*γ* are also capable of regulating gene expression independently of binding to PPREs. The activity of a number of transcription factors, for example NF-*κ*B (nuclear factor-*κ*B), AP-1 (activator protein-1), and STAT-1 (signal transducer and activator of transcription), can be inhibited by PPARs either via direct interaction or by competition for limited supplies of coactivators [11, 13]. This function is important in regard to the anti-inflammatory properties of PPARs since proinflammatory gene expression is mainly affected by this direct repression of transcription factors.

Since their first description, PPARs have been implicated in numerous biological processes and diseases. They play a central role in the regulation of glucose, lipid, and lipoprotein metabolism, and PPAR agonists are established drugs for treatment of diabetes (glitazones) and dyslipidemia (fibrates) [14]. Furthermore, angiogenesis, cellular differentiation and proliferation as well as apoptosis are mediated by PPARs, so that PPARs were suggested to contribute to tissue repair and cancer-related pathways [15, 16]. More recent studies could demonstrate an additional involvement of PPARs in senescence-related diseases, in the regulation of male and female fertility, and in cardiovascular conditions like atherosclerosis [17, 18]. 

In regard to the immune system, PPARs have been identified as crucial regulators of inflammatory gene expression. Various immune cells were shown to express PPARs including dendritic cells, monocytes, macrophages, B- and T-lymphocytes, and vascular endothelial cells [11]. At the site of inflammation, PPAR*β*/*δ* ligands reduce the expression of adhesion molecules by endothelial cells and decrease the release of cytokines and chemokines by macrophages. PPAR*α* activation could be linked to inhibited production of proinflammatory cytokines from TH1 cells and increased release of anti-inflammatory cytokines from TH2 cells [19]. These numerous anti-inflammatory properties of PPARs have suggested a central role of PPAR activation in attenuating the inflammatory response after trauma and tissue injury.

## 3. The Role PPARs in Central Nervous System Trauma

Traumatic injury of the CNS remains a major health problem worldwide and represents one of the leading causes of death and persisting neurological impairment in industrialized countries [1, 20]. Brain damage after traumatic brain injury (TBI) is determined by a combination of primary and secondary insults [21, 22]. While the primary injury is induced by the mechanical impact to the skull and brain, secondary brain injury results from an uncontrolled neuroinflammatory response within the injured brain. 

Recent experimental studies have indicated that activation of PPARs might represent a promising therapeutic strategy for counteracting this deleterious posttraumatic inflammation [23, 24]. PPAR expression has been described in intracerebral and spinal vascular endothelial cells, neurons, astrocytes, and oligodendrocytes [25, 26]. Interestingly, two recent studies could show an upregulation of cortical PPAR expression both in experimental models of CNS injury, and in the brain of head-injured patients [27–29]. 

**Figure 1:**
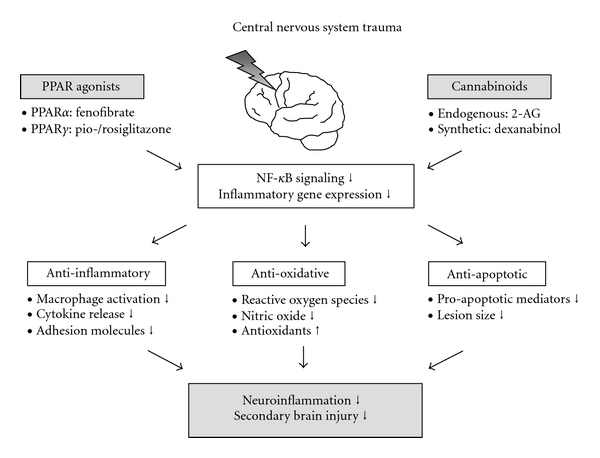
Overview of the anti-inflammatory and neuroprotective effects of selected PPAR agonists in central nervous system (CNS) injury, as exemplified in the setting of traumatic brain injury. See text for detailed explanations. Abbreviations: *PPAR*, *peroxisome proliferator-activated receptor*; *2-AG*, *2-arachidonyl glycerol*; *NF-*κ*B*, *nuclear factor-*κ*B*; *CNS*, *central nervous system*.

The key role of PPARs in attenuating neuroinflammation has been examined in multiple models of stroke, ischemia/reperfusion injury and CNS trauma as well as in chronic brain disorders like Parkinson's and Alzheimer's disease [6, 30]. In these studies [30–32], PPAR activation was shown to induce neuroprotection through three distinct main mechanisms, as outlined schematically in Figure 1. First, the cerebral inflammatory response itself was modulated by PPAR-induced inhibition of macrophage/monocyte activation and proinflammatory cytokine release and suppressed upregulation of cellular adhesion molecules [33, 34]. Second, PPARs were able to modulate oxidative stress in the brain, a significant component enhancing the inflammatory cascade and secondary brain injury; activation of PPARs led to reduced production of reactive oxygen species and nitric oxide and increased the release of antioxidants around the injured tissue [35, 36]. Finally, PPARs have been shown to delay neuronal apoptosis and decrease lesion sizes by the inhibition of proapoptotic mediators [27, 37].

As mentioned above, PPARs act by repressing central inflammatory transcription factors like NF-*κ*B or AP-1 [11]. This mechanism is likely to underlie the described anti-inflammatory effect of PPAR activation after CNS trauma and seems to be responsible for the reduced expression of several key downstream inflammatory genes in the injured CNS [32]. 

Genovese et al. have examined the effect of PPAR-*α* gene depletion in a mouse model of spinal cord injury: PPAR-*α* ^−/−^ mice showed significantly augmented injury parameters such as edema, neutrophil infiltration, and apoptosis [38]. Moreover, the therapeutic effect of glucocorticoids has been compared in PPAR-*α* ^−/−^ and wild-type mice [39]. Since the anti-inflammatory properties of glucocorticoids were markedly weakened in PPAR-*α* ^−/−^ mice, it was hypothesized that PPAR-*α* could contribute to the anti-inflammatory activity of glucocorticoids in CNS trauma [39].

Paying attention to the above mentioned crucial role of PPARs in neuroprotection, pharmacological agonism to PPARs has been of particular interest to the neuroscience community in recent years (Figure 1) [33, 40]. Originally, PPAR-*α* ligands have been described as highly promising anti-inflammatory drugs (reviewed in [6]). In a rat model of traumatic brain injury, the PPAR-*α* agonist fenofibrate was revealed to significantly ameliorate pathophysiology after injury including an improvement of neurological scores and a reduction of brain edema [41]. In addition, fenofibrate showed antioxidant effects demonstrated by decrease of intracerebral markers of oxidative stress [36]. A study from the same group assessed a synergistical effect of a combination therapy with fenofibrate and simvastatin, a lipid-lowering drug [42]. The administration of these pharmacological compounds in combination after TBI exerted a more sustained neurological recovery than the monotherapies and might have important significance for the treatment of TBI [42].

More recently, studies have moved their focus on the therapeutic efficacy of agonists to PPAR-*γ* [24, 43]. Pioglitazone and rosiglitazone are both approved drugs for diabetes treatment and have been shown to bind to PPAR-*γ* [24]. Sauerbeck et al. hypothesized that pioglitazone would promote neuroprotection in a rat controlled cortical impact model of TBI where the drug was administered every 24 hours beginning 15 minutes after injury [44]. Treatment with pioglitazone resulted in significantly improved cognitive function, reduced lesion size, and prevented the activation of microglia, compared to the vehicle-treated animals [44, 45]. Similar beneficial effects could be demonstrated for rosiglitazone both in traumatic brain and spine injury [27, 34, 46, 47]. Taken together, PPAR-*γ* agonists have been shown to exert a wide range of anti-inflammatory, anti-oxidative, and anti-apoptotic effects and were like that able to counteract the main pathophysiological events occurring in the development of secondary CNS injury [27, 44, 47–49]. These observations could strengthen the idea that the field of application of PPAR-*γ* agonists could be expanded from antidiabetic drugs to therapeutic agents counteracting neuroinflammation and improving the outcome of patients with traumatic CNS injuries.

 In addition to synthetic PPAR agonists, a protective role of cannabinoids on the sequelae of CNS trauma has been suggested in recent years [50–52]. The group of cannabinoids consists of endogenous ligands, such as 2-arachidonyl glycerol (2-AG), and synthetic drugs like dexanabinol (HU-211) [53]. After TBI in mice, elevated levels of 2-AG could be detected [54]. Administration of 2-AG to mice after experimental head injury suppressed the release of reactive oxygen species and proinflammatory cytokines and improved clinical recovery [54, 55]. Similar to that, the synthetic cannabinoid HU-211 revealed as a cerebroprotectant attenuating breakdown of the blood-brain barrier and production of cytokines [56, 57]. Recent studies have provided evidence that an increasing number of anti-inflammatory actions of cannabinoids are not mediated by the classical cannabinoid receptors but are induced by agonistic action of cannabinoids to PPARs [53, 58]. Oleylethanolamide that is structurally related to the endogenous cannabinoid anandamide was found to bind to PPAR-*α*, and Δ^9^-tetrahydrocannbinol (THC), the active ingredient of cannabis, has been described as a PPAR-*γ* ligand [59, 60]. Interestingly, the mechanism by which 2-AG suppresses interleukin-2 expression was linked to targeting PPAR-*γ* and was independent of cannabinoid receptors [58]. Due to the promising anti-inflammatory properties of cannabinoids, a randomized phase II and III clinical trial was initiated that aimed to test HU-211 in the treatment of brain trauma [52, 61, 62]. In the study patients treated with HU-211 achieved significantly decreased intracranial pressure, and a trend towards better neurological outcome was observed [61].

 In summary, numerous attempts of targeting PPARs both by cannabinoids and by synthetic PPAR ligands have revealed the huge neuroprotective ability of PPAR agonism. Further studies will have to examine if administration of a single PPAR ligand or a combination of cannabinoids with synthetic PPAR agonists might have the potential to be introduced in the clinic of traumatic CNS injury.

## 4. PPARs in Ischemia/Reperfusion Injury

Ischemia/reperfusion (I/R) injury represents a challenging pathophysiological condition with serious clinical implications, in a broad field of conditions such as organ transplantation, compartment syndrome, myocardial infarction, stroke, and hemorrhagic, traumatic, or septic shock [5, 63]. Tissue ischemia together with subsequent reperfusion has been shown to trigger a whole cascade of inflammatory events that, if not counteracted in early stages, result in cell necrosis with irreversible tissue damage in affected organs [7]. Research efforts in recent years have provided increasing evidence that PPARs represent major regulators of this inflammatory response; PPAR activation could be shown to restrict inflammation and exert multiple beneficial effects against ischemia/reperfusion injury [7, 15]. Consequently, pharmacological agents targeting PPARs have been suggested as potential therapeutics for the treatment of I/R.

Similar as described for traumatic CNS injuries, a strong relationship between PPAR tissue expression and I/R injury could be demonstrated. In kidney I/R, PPAR*γ* expression was strongly increased in endothelial cells, interstitial cells, and collecting ducts of the kidney peaking from 1.5 to 5 hours after reperfusion [64, 65]. Similar upregulation of PPAR*γ* was detected in the cortical peri-infarct area after focal cerebral ischemia in rats [66]. Interestingly, Lee and colleagues have recently found in a model of transient cerebral ischemia that PPAR*γ*-immunoreactivity in the hippocampus was colocalized with microglial cells indicating a high functional state of microglia in the ischemic brain [67]. 

**Figure 2:**
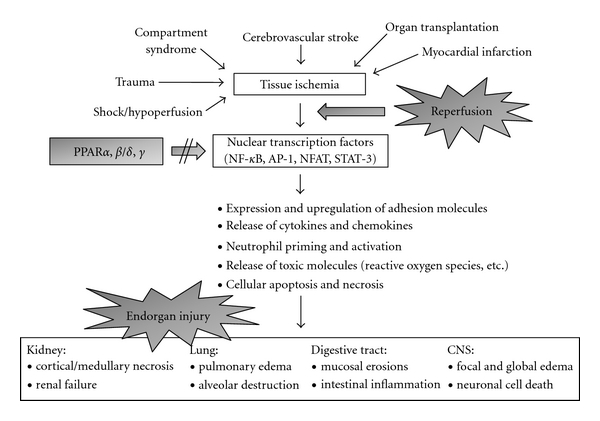
Schematic depiction of the inflammatory events occurring during the pathophysiology of ischemia/reperfusion (I/R) injury, and potential pharmacological effects of PPAR ligands, by inhibition of nuclear transcription factors. See text for detailed explanations. Abbreviations: *PPAR*, *peroxisome proliferator-activated receptor*; *CNS*, *central nervous system*; *NF-*κ*B*, *nuclear factor-*κ*B*; *AP-1*, *activator protein-1*; *NFAT*, *nuclear factor of activated T cells*; *STAT-3*, *signal transducer and activator of transcription-3*.

In recent years, animal studies of I/R injury in various organs have revealed a crucial role of PPARs in reducing or even preventing tissue injury and organ dysfunction after ischemia and reperfusion [15, 68, 69]. Consequently, a wide variety of natural and synthetic PPAR agonists was tested in experimental I/R models and was shown to significantly improve the outcome of I/R injury [5, 70, 71]. The mechanisms of tissue protection by PPAR ligands have been thought to be multifactorial [7], since these agonists can interact with variable parameters of the IR-induced inflammatory cascade and inhibit multiple targets on the way to injury progression, as outlined in Figure 2. The proposed mechanisms of action include: (i) reduced expression of adhesion molecules like ICAM-1 and p-selectin on endothelial cells [72, 73], (ii) decreased vascular permeability with suppressed edema formation [74], (iii) inhibited release of proinflammatory mediators like cytokines and chemokines [68, 75], (iv) reduced activation of inflammatory cells like neutrophils [72, 76], (v) decreased formation of reactive oxygen species [77, 78], (vi) suppressed cell apoptosis and necrosis [79, 80], and (vii) inhibited platelet aggregation and thrombus formation [81]. Similar as described for CNS injuries, the majority of these anti-inflammatory effects is initiated by PPAR-induced suppression of transcription factors (mainly NF-*κ*B) and subsequent inhibition of proinflammatory gene transcription (Figure 2) [35, 69, 70, 80]. 

In addition to the mentioned general effects of PPAR activation on the inflammatory response in I/R (Figure 2), numerous tissue-specific impacts of PPAR agonists have been described in different organ systems (reviewed in [5, 15]).

### 4.1. Renal I/R

Renal ischemia is a major cause of acute renal failure that is complicated by the fact that subsequent reperfusion of hypoxic tissue may cause additional injury [82]. Agonists to all three PPAR isoforms, PPAR*α*, PPAR*β*/*δ*, and PPAR*γ*, significantly reduced tissue damage in mice subjected to kidney ischemia and reperfusion [77, 83, 84]. This renoprotection was reflected in attenuation of cortical and medullary necrosis, reduction of histological signs of renal damage, and finally in strongly increased renal function with lowered serum creatinine and urea nitrogen levels [69, 85, 86]. The mechanisms underlying these beneficial properties may consist in induction of fatty acid *β*-oxidation enzymes by PPARs in kidney tissue; transgenic mice expressing PPAR*α* in the proximal tubule were shown to exert increased fatty acid oxidation and were protected from I/R-induced kidney failure [87, 88].

### 4.2. Pulmonary I/R

I/R injury of the lung still occurs in 20% of patients after lung transplantation and remains the main cause of death during the first month after transplantation [89]. Application of the synthetic PPAR*γ* ligand pioglitazone or the natural PPAR*γ* agonist 15-deoxy-Δ^12,14^-prostaglandin J2 (15d-PGJ2) before ischemia could attenuate lung I/R injury in rats [70, 74]. A recent study of Okada et al. indicated that PPAR*γ* activation suppresses activation of the zinc finger transcription factor early growth response gene-1 (Egr-1) that has a crucial role in the inflammatory response in ischemic vessels [70]. Thus, as a consequence of PPAR*γ* activation, the induction of Egr-1 target genes such as interleukin-1*β* is prevented, IR-associated leukostasis is decreased, and overall survival is improved [70].

### 4.3. Gastrointestinal I/R

Intestinal and gastric I/R injuries are serious clinical conditions resulting from abdominal aneurism, acute mesenteric ischemia, small bowel transplantation, or shock [90]. In rodent models of intestinal I/R, all three isotypes of PPAR agonists showed profound anti-inflammatory, anti-oxidative and anti-apoptotic effects that were associated with a decreased I/R-induced mortality rate [68, 72, 80]. Similar to that, pioglitazone and rosiglitazone suppressed gastric mucosal erosion and damage in gastric I/R rats [78, 91]. Additionally, beneficial effects of early enteral nutrition after gut I/R could be linked to PPAR induction. The nutrition component glutamine was reported to exert gut protection by activation of PPAR*γ* [92, 93].

### 4.4. Ischemic Brain Injury

Ischemic cerebrovascular disease represents the third leading cause of death and is one of the major causes of neurological dysfunction and disability [94]. Various studies have suggested that PPAR agonists may prevent or decrease the severity of both focal and global ischemia [66, 95]. In humans, stroke incidence was reduced when men with coronary heart disease and low HDL and LDL cholesterol values were treated with the fibrate and PPAR*α* agonist gemfibrozil [96]. Application of PPAR*α*, PPAR*β*/*δ*, and PPAR*γ* ligands in transient ischemic brain injury of rodents resulted in significantly attenuated neuronal damage and reduced infarction volume, increased cerebral blood flow, and improved neurological outcome parameters [71, 97–99]. This neuroprotection was observed when animals were treated preventively before ischemia, at the time of cerebral infarction, or shortly after that with a time window of efficacy of two hours after ischemia [73, 76, 100]. In contrast to transient ischemia, PPAR*γ* activation failed to decrease infarction volume when the blood flow was interrupted permanently without subsequent reperfusion [101–103]. These findings support evidence that the neuroprotective role of PPAR*γ* is specific to events occurring during reperfusion. 

Overall, various studies provide evidence that ligands to PPARs cause a substantial reduction of I/R injury in diverse organs by interfering with multiple targets of the I/R-induced inflammatory cascade.

## 5. PPARs in Sepsis and Shock

Shock and sepsis are serious complications of severely ill patients in intensive care units that can ultimately lead to multiple organ failure and death. There is now increasing evidence that the immune response following severe trauma is divided into two phases [4]. The early hyperinflammatory state is characterized by an overactivation of the innate immune system with increased priming of neutrophils and extensive release of inflammatory mediators like cytokines or reactive oxygen species. Often this first period is followed by a second immunosuppressive phase associated with the attenuation of adaptive immunity and decreased T cell function [3, 8].

 The role of PPAR activation in modulating this posttraumatic immune response is ambiguous and has been described as “double-edged sword” [8]. Numerous preclinical studies have supported central beneficial effects of PPAR activation in the first proinflammatory period [104–107]. On the other hand, PPARs have been shown to exert proapoptotic and desensitizing characteristics on inflammatory cells what might increase the susceptibility of the trauma patient to secondary infections during the immunosuppressive phase [108, 109].

 The protective properties of PPAR agonists have been examined in multiple animal models of sepsis and shock (Table 1) (reviewed in [8, 12]). Using intraperitoneal LPS injection as a model for endotoxic shock in mice, Kaplan et al. could demonstrate that endotoxin-induced inflammation was associated with reduced expression of PPAR*γ* and with activation of NF-*κ*B in the lung [104]. Treatment with the natural PPAR*γ* ligand 15d-PGJ2 significantly improved survival rate and attenuated inflammation signs by repressing activation of NF-*κ*B and enhancing the expression of cytoprotective heat shock protein in the lung [104]. Similar to that biomarkers of liver and kidney injury and inflammatory cytokines were suppressed when PPAR*γ* agonists were applied before induction of endotoxic shock [110]. 

In hemorrhagic shock in rats, the PPAR*γ* agonists 15d-PGJ2 and ciglitazone ameliorated mean arterial pressure, blunted neutrophil activation, and abolished dysfunction of kidney, liver, lung, and intestine [111, 112]. These effects were mediated through inhibition of the NF-*κ*B pathway. Moreover, two recent studies have found that hemorrhaghic shock-induced apoptosis in the liver and the lung can be decreased by ciglitazone treatment [105, 113]. Herein, attenuation of lung apoptosis was associated with significant reduction in caspase-3 activity and increased phosphorylation of the prosurvival kinase Akt [113].

 In a rodent model of polymicrobial sepsis, both PPAR*γ* and PPAR*β*/*δ* activation limited the extent of organ dysfunction caused by cecal ligation and puncture (CLP) [106, 114]. PPAR*β*/*δ*-deficient mice suffered exaggerated lethality when subjected to CLP and exhibited severe lung injury and higher levels of TNF*α* [106, 115]. Application of a PPAR*β*/*δ* agonist significantly improved survival in polymicrobial sepsis by a mechanism that might involve activation of Akt, inhibition of the MAPK-ERK1/2-signaling pathway, and subsequent suppression of NF-*κ*B activity [106]. 

Multiple organ failure as a final consequence of severe shock and sepsis can be induced in mice by intraperitoneal administration of zymosan. When animals were treated with a PPAR*γ* or PPAR*β*/*δ* agonist after zymosan injection, peritoneal exude formation and neutrophil infiltration were reduced, and lung, liver and pancreatic injury, and renal dysfunction were attenuated [116, 117]. 

Besides of the classical PPAR agonists, endogenous factors have recently been shown to exert immunomodulatory properties by activating PPARs in different shock models. C-peptide is a 31-amino acid peptide cleaved from insulin during insulin synthesis that has been considered to have minimal biological activity [118]. However, in vitro and in vivo studies have now reported that C-peptide may stimulate PPAR*γ* and thus modulate the inflammatory response in ischemia/reperfusion and shock [119–121]. A study of Vish et al. could demonstrate that treatment with C-peptide improved survival rates and reduced plasma levels of cytokines when mice were subjected to endotoxic shock [121]. C-peptide also upregulated nuclear expression of PPAR*γ* and reduced phosphorylation of ERK-1 and -2 [121]. Moreover, in a model of hemorrhagic shock in rats, hypotension and lung inflammation were significantly ameliorated after C-peptide infusion what was associated with reduced expression of AP-1 and NF-*κ*B and activation of PPAR*γ* [122].

 Similarly, the vasoactive hormone adrenomedullin has been shown to be beneficial in sepsis by abrogating the progression to irreversible shock. In addition to its vasodilatory function, adrenomedullin decreases cytokines in the circulation of septic animals what synergistically protects animals from dying of sepsis [123]. These latter anti-inflammatory effects seem to be mediated by mechanisms involving intracellular cAMP increase, followed by upregulation of PPAR*γ* and subsequent suppression of cytokine release [124]. 

In contrast to the multiple beneficial properties of PPAR agonism, recent studies have revealed additional effects of PPAR activation on immune cells that might compromise the role of these cells in host defense. Neutrophils represent the central cellular component in the sepsis-induced innate immune response and were previously shown to express PPAR*γ* [125]. Treatment of isolated neutrophils with PPAR*γ* agonists resulted in a significant reduction of neutrophil chemotactic activity in vitro [109]. When sepsis was induced in mice, chemotaxis of neutrophils was suppressed compared to healthy mice, but treatment with a PPAR*γ* antagonist restored chemotactic activity to control levels [109]. Since neutrophil expression was increased in septic patients and mice, the authors of the study suggested that the inhibited migration of neutrophils during sepsis might occur as a result of PPAR*γ* activation.

Similar to neutrophils, upregulation of PPAR*γ* was observed in T cells of septic patients [108]. These cells responded with apoptosis when they were exposed to PPAR*γ* agonists [108]. In an animal model of sepsis, sepsis-induced T cell depletion was abrogated after application of a PPAR*γ* antagonist [126]. Consequently, a pivotal involvement of PPAR*γ* in T cell apoptosis was hypothesized what might contribute to lymphocyte loss and breakdown of defense mechanisms during sepsis. 

Taken together, there is increasing evidence that PPAR activation has multiple anti-inflammatory properties in shock, sepsis, and multiple organ failure. However, PPAR*γ* agonism was also found to contribute to function loss of neutrophils and depletion of lymphocytes what might be deleterious in the immunosuppressive phase of sepsis. This fact has to be taken in consideration when a potential therapeutical use of PPAR agonists in the treatment of shock and sepsis is discussed.

## 6. The Role of PPARs in Wound Healing Processes

After skin trauma and injury, wound healing is a life-saving process during which the wound bed is covered with a new protective epithelium. This healing process is mainly divided into three phases. The initial inflammatory stage is followed by proliferation and migration of keratinocytes with reepithelialization of the wound [19]. In parallel, dermal repair involves activation and proliferation of fibroblasts and angiogenesis (remodeling phase) what finally results in wound closure.

 While PPAR expression progressively disappears from the interfollicular epidermis after birth, PPAR*α* and PPAR*β*/*δ* are reactivated in keratinocytes at the wound edges of damaged skin [127]. Hereby, the upregulation of PPAR*α* is transient and correlates with the early inflammatory phase of wound healing. A study of Michalik et al. could demonstrate that PPAR*α* ^−/−^ mice exert a transient delay of wound healing during the first 4 days after injury coinciding with increased PPAR*α* expression during the inflammatory stage [127, 128]. Furthermore, both the recruitment of neutrophils and monocytes was impaired in PPAR*α* ^−/−^ mice. In contrast to PPAR*α*, PPAR*β*/*δ* expression persists throughout the entire repair process and is correlated to keratinocyte proliferation, adhesion, and migration in order to reepithelialize the wounded area. Consistent to that, wound healing of PPAR*β* ^+/−^ mice was delayed during the whole repair process, and final wound closure was postponed by two to three days [127]. 

**Figure 3:**
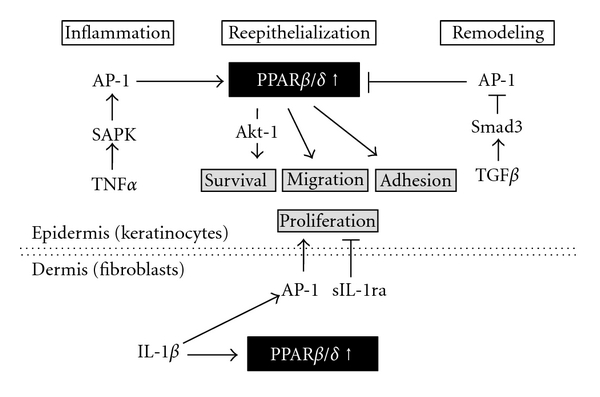
Role of PPAR*β*/*δ* expression in the wound healing processes after tissue injury. See text for detailed explanations. Abbreviations:* PPAR*, *peroxisome proliferator-activated receptor*; *TNF*α**, *tumor necrosis factor *α**; *SAPK*, *stress-associated protein kinase*; *AP-1*, *activator protein-1*; *PPAR*, *peroxisome proliferator-activated receptor*; *TGF*β**, *transforming growth factor *β**; *IL-1*β**, *interleukin-1 *β**; *sIL-1ra*, *soluble interleukin-1 receptor antagonist*.

As outlined in Figure 3, the expression of PPAR*β*/*δ* during wound healing is characterized by a specific time pattern and is tightly regulated by signaling cascades, providing both stimulatory and negative feedback mechanisms [18, 129]. Upon injury, proinflammatory cytokines like TNF*α* activate the stress-associated protein kinase pathway and induce AP-1 binding to the PPAR*β*/*δ* promotor, leading to the stimulation of PPAR*β*/*δ* expression [130]. In the healing process, PPAR*β*/*δ* represses apoptosis cascades through transcriptional upregulation of integrin-linked kinase (ILK) and 3-phosphoinositide-dependent kinase-1 (PDK), and consequent Akt-1 activation [131]. The resulting resistance to cell death helps to maintain a sufficient number of viable keratinocytes for reepithelialization. Moreover, PPAR*β*/*δ* is implicated in keratinocyte adhesion and migration of keratinocytes, two key processes during reepithelialization [131, 132]. After the inflammatory and early reepithelialization phase, during which PPAR*β*/*δ* expression is maximal, PPAR*β*/*δ* is progressively reduced in the epithelium. The responsible antagonization process is initiated by TGF*β*-1/Smad3-mediated inhibition of AP-1 binding to the PPAR*β*/*δ* promotor in the late reepithelialization/remodeling phase, resulting in downregulation of the PPAR*β*/*δ* gene [133, 134]. Taken together, the healing process represents a delicate balance between early proinflammatory signals triggering PPAR*β*/*δ* expression and negative feedback pathways at later stages of wound healing. Since both processes temporally overlap, an intensive crosstalk between the two signaling pathways is suggested that contribute to fine-tuning of wound closure [133].

 In addition to the described anti-apoptotic properties, PPAR*β*/*δ* also influences keratinocyte differentiation and proliferation by to-date unknown mechanism. Chong et al. could recently demonstrate that PPAR*β*/*δ*-dependent interplay between the epidermis and dermis is essential for controlling epidermal proliferation (Figure 3) [18, 135]. They found that interleukin-1 (IL-1) produced by keratinocytes stimulates AP-1 transcription factor in dermal fibroblasts and consequently increases the production of mitogenic factors that enhance keratinocyte proliferation. In parallel, IL-1 activates PPAR*β*/*δ* expression in fibroblasts, which increases the production of the secreted IL-1 receptor antagonist (sIL-1ra), a protein inhibiting IL-1 signaling. As a consequence, the IL-1-induced production of mitogenic factors by fibroblasts is reduced, and keratinocyte proliferation is decreased when wound closure comes to an end [135]. This study provides evidence that PPAR*β*/*δ* participates in regulating epidermal proliferation via a paracrine mechanism.

 In summary, efficiency in the wound healing process is guaranteed both by interactions within the epidermis as well as by epithelial-mesenchymal crosstalk. Thus, keratinocyte differentiation and survival are provided in the early healing process and, at the same time, exaggerated proliferation and hypertrophic scarring are avoided in later stages. It has been suggested that these insights into the coordination of wound healing might contribute to develop better treatment strategies for chronic wound disorders [18].

## 7. Conclusion

In summary, various studies have provided evidence that PPARs have a substantial impact on reducing the extent of post-injury inflammation after major trauma, mainly by the suppression of the central transcription factor NF-*κ*B. These anti-inflammatory properties could be supported by the use of synthetic and natural PPAR ligands in multiple animal models of CNS trauma, I/R injury, and shock. Furthermore, PPAR activation was found to be responsible for the balance between differentiation, cell survival, and apoptosis during wound healing. Synthetic PPAR ligands are readily available from clinical trials in diabetes and hyperlipidemia. The basic science studies described in this review article provide a strong rationale for testing these pharmacological compounds for their anti-inflammatory potential in the clinical setting of posttraumatic hyperinflammation in the near future.

## Figures and Tables

**Table 1:** Selected publications on experimental studies testing the anti-inflammatory effects of PPAR ligands in various models of sepsis and shock.

Pathological condition	PPAR isotype/ligand	Ligand-induced effects	Affected signaling pathway	Reference
Endotoxic shock	PPAR*α*/fenofibrate	Decrease in coagulation activation (monocyte tissue factor expression), protection against endothelial dysfunction	Not examined	Wiel et al. [107]
	PPAR*γ*/rosiglitazone	Suppression of biomarkers for liver and kidney injury and of cytokines, inhibition of heart rate increase	Not examined	Wu et al. [110]
	PPAR*γ*/15d-PGJ2	Improvement of survival rate, reduction of adhesion molecule expression, and of neutrophil infiltration in tissues	NF-*κ*B, HSP (heat shock protein) 70	Kaplan et al. [104]

Hemorrhagic shock	PPAR*γ*/ciglitazone	Amelioration of mean arterial pressure, reduction of plasma cytokine levels, decrease of apoptosis in lung and liver	NF-*κ*B	Chima et al. [112]
			Caspase-3, PI3/Akt	Chima et al. [113], Zingarelli et al. [105]
	PPAR*γ*/15d-PGJ2	Attenuation of renal dysfunction and of liver, lung, and intestine injury	Not examined	Abdelrahman et al. [111]

Polymicrobial sepsis/septic shock	PPAR*β*/*δ*	Decrease in cytokine release, attenuation of organ dysfunction, reduced expression of inducible nitric oxide synthase	Akt, GSK-3*β*, ERK1/2, STAT-3, NF-*κ*B	Kapoor et al. [106]
				Zingarelli et al. [115]
	PPAR*γ*/ciglitazone and 15d-PGJ2	Amelioration of hypotension and survival, decreased inflammatory signs in lung, colon, and liver	NF-*κ*B, AP-1	Zingarelli et al. [114]

Multiple organ failure	PPAR*β*/*δ*	Reduction of peritoneal exsudate formation and of neutrophil infiltration, attenuation of multiple organ dysfunction syndrome	Not examined	Galuppo et al. [117]
	PPAR*γ*/rosiglitazone	Attenuation of peritoneal exsudation and of organ injury and dysfunction	Not examined	Cuzzocrea et al. [116]
